# Stereoselective Property of 20(*S*)-Protopanaxadiol Ocotillol Type Epimers Affects Its Absorption and Also the Inhibition of P-Glycoprotein

**DOI:** 10.1371/journal.pone.0098887

**Published:** 2014-06-02

**Authors:** Wenyan Wang, Xiangmeng Wu, Li Wang, Qingguo Meng, Wanhui Liu

**Affiliations:** School of Pharmacy, Yantai University, Yantai, Shandong, China; University of East Anglia, United Kingdom

## Abstract

Stereoselectivity has been proved to be tightly related to drug action including pharmacodynamics and pharmacokinetics. (20S,24R)-epoxy-dammarane-3,12,25-triol (24R-epimer) and (20S,24S)-epoxy-dammarane-3,12,25-triol (24S-epimer), a pair of 20(S)-protopanaxadiol (PPD) ocotillol type epimers, were the main metabolites of PPD. Previous studies have shown that 24R-epimer and 24S-epimer had stereoselectivity in pharmacological action and pharmacokinetics. In the present study, the aim was to further study the pharmacokinetic characteristics of both epimers, investigate their absorption mechanism and analyze the selectivity effects of ocotillol type side chain and C24 stereo-configuration on P-glycoprotein (P-gp) *in vivo* and *in vitro*. Results showed that the absolute bioavailability of 24R-epimer was about 14-fold higher than that of 24S-epimer, and a linear kinetic characteristic was acquired in doses of 5–20 mg/kg for both epimers after oral administration. Furthermore, the apparent permeability coefficients of 24R-epimer were 5–7 folds higher than that of 24S-epimer having lower efflux ratios in Caco-2 cell models. Moreover, both 24R-epimer and 24S-epimer had similar inhibitory effects on P-gp by increasing cellular retention of rhodamine 123 in Caco-2 cells and decreasing efflux of digoxin across Caco-2 cell monolayers. In situ *in vivo* experiments showed that the inhibition of 24R-epimer on P-gp was stronger than that of 24S-epimer by single-pass intestinal perfusion of rhodamine 123 in rats. Western blot analyses demonstrated that both epimers had no action on P-gp expression in Caco-2 cells. In conclusion, with respect to the stereoselectivity, C24 S-configuration of the ocotillol type epimers processed a poor transmembrane permeability and could be distinguished by P-gp. Sharing a dammarane skeleton, both 24R-epimer and 24S-epimer were potent inhibitors of P-gp. This study provides a new case of stereoselective pharmacokinetics of chiral compounds which contributes to know the chiral characteristics of P-gp and structure-action relationship of PPD type and ocotillol type ginsenosides as a P-gp inhibitor.

## Introduction

**Figure 1:**
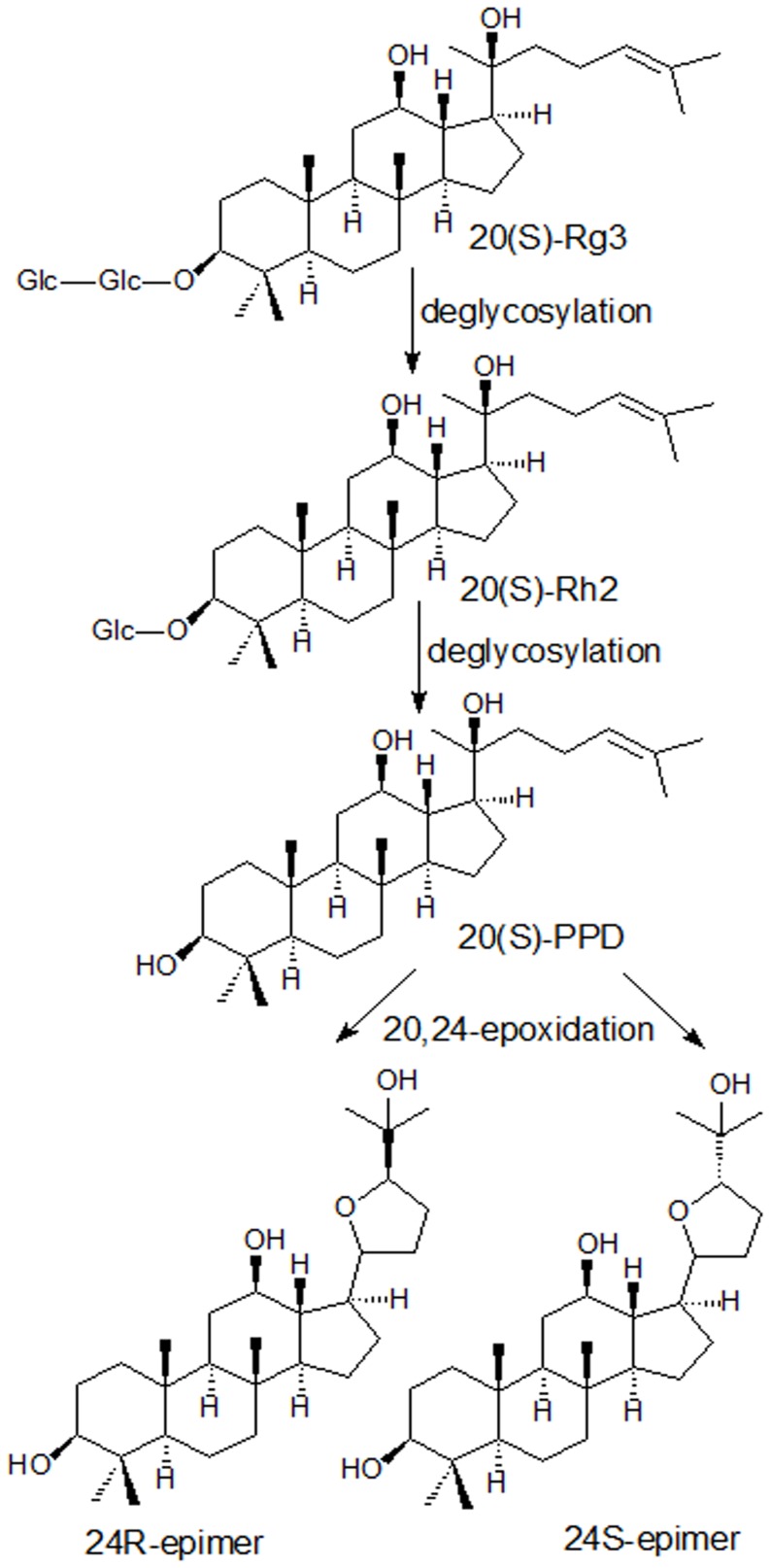
Proposed possible metabolism pathway of ginsenoside Rg3 and its deglycosylated metabolites.

Ginseng, reputed as the king of herbs, has a wide range of therapeutic applications. It has been revealed that the main active ingredients of ginseng are ginsenosides [1]. Ginsenosides have a four-ring, a steroid-like structure with sugar moieties attached, and about 80 different forms have been isolated and identified from various ginseng drugs, which was subdivided into four classes according to aglycones: 20(S)- protopanaxadiol (PPD) type, 20(S)-protopanaxatriol (PPT) type, oleanic type and ocotillol type [2]–[3]. Ginsenoside Rg3, a kind of PPD type ginsenosides, exerts many pharmacological activities such as tumor-suppressing, antimetastatic, hepatoprotective and neuroprotective effects [4]–[8]. Studies showed that ginsenoside Rg3 could transform to ginsenoside Rh2 and further deglycosylated to PPD [9]. Both Rh2 and PPD also have shown anticancer activity and synergy with chemotherapy drugs comparable to or higher than that of Rg3 [10]–[13]. The oral bioavailability of ginsenosides Rg3 and Rh2 was less than 5% [14], [15]. The oral bioavailability of PPD was improved but still low (31.0∼36.8% in rats and 9.6% in dogs) [16]. The studies demonstrated 20,24-epoxides, (20S,24R)-epoxy-dammarane-3,12,25-triol (24R-epimer) and (20S,24S)-epoxy- dammarane-3,12,25-triol (24S-epimer), were main metabolites of PPD [16], [17]. And they were a pair of ocotillol type epimers. The proposed metabolism route of ginsenosides Rg3 was shown in Fig. 1. It is obviously that here're many chiral carbons in the molecule structure, for example, C3, C12 and C20, etc. Particularly, the ocotillol type metabolites of PPD have a new chirality C24.

Many researchers paid close attention to C20 stereo-configuration of ginsenosides, reported the different pharmacological effects of stereoisomers produced from the chirality C20. The peroxisome proliferator activated receptor-γ activity of 20(S)-Rg3 is 10-fold higher than that of 20(R)-Rg3 [18]. 20(R)-Rg3 has more potent activity than 20(S)-Rg3 in stimulating the immune response [19]. 20(S)-Rh2 inhibited the proliferation of both androgen-dependent and -independent prostate cancer cells, while 20(R)-Rh2 not [20]. 20(R)-Rh2 was a selective osteoclastogenesis inhibitor with no apparent cytotoxicity [21]. Meanwhile, these epimers of ginsenosides also showed stereoselectivity in pharmacokinetics. The study [9] reported the transformation amount of 20(S)-Rg3 into 20(S)-Rh2 or 20(S)-PPD in human fecal microflora was 19-fold higher than that of 20(R)-Rg3 into 20(R)-Rh2 or 20(R)-PPD. After oral administration, S-configuration of Rg3, Rh2 and its further deglycosylation metabolite PPD exhibited significantly higher plasma concentrations than the one with R-configuration [22], [23]. These indicated that the metabolism and absorption of the ginsenosides with S-configuration at C20 had stereoselective superiority.

However, the different pharmacological effects of stereoisomers produced from the chirality C24 of ocotillol type ginsenosides were hardly reported compared to C20. In the pharmacological study, 24R-epimer exerted cardioprotective effects similar with PPD, but 24S-epimer not [24]. Moreover, our previous study demonstrated there were pharmacokinetic differences between 24R and 24S epimers [25]. After oral administration at an equal dose, the AUC of 24R-epimer was 21-fold higher than that of 24S-epimer. This may suggest that the stereo-configuration of ocotillol side chain was linked to the chirality recognition and selectivity of ocotillol type triterpene saponins *in vivo*. The membrane permeability is an important factor influencing drug absorption. Moreover, P-glycoprotein (P-gp), a member of drug transporters, often was a barrier for drug absorption. And as biomacromolecules, P-gp owns the ability to stereoselectively distinguish the ligands [26]. Many studies have been reported that ginsenosides Rg3, Rh2 and PPD were all able to inhibit P-gp activity and they were not substrates of the P-gp [27]–[29]. The ocotillol type empiric metabolites of PPD share a C20 S-configuration and dammarane skeleton of PPD, and have a new chirality C24. Therefore, it is worth further study whether ocotillol type side chain or C24 stereo-configuration was linked to the chirality recognition and selectivity effect to P-gp *in vivo* and *in vitro*, and to analyze the potential drug-drug interactions related to drug transporters. This study provides a new case to know chiral recognition of P-gp and structure-action relationship of PPD type and ocotillol type ginsenosides as a P-gp inhibitor.

## Materials and Methods

### Materials

(20S,24R)-epoxy-dammarane-3,12,25-triol (24R-epimer) (purity >98%) and (20S, 24S)-epoxy-dammarane-3,12,25-triol (24S-epimer) (purity >98%) were obtained from the Department of Medical Chemistry of Yantai University (Yantai, China). Rhodamine 123 was purchased from Sigma-Aldrich (St. louis, MO). Digoxin and verapamil were purchased from the Chinese National Institute for the Control of Pharmaceutical and Biological Products (Beijing, China). Fluorescein disodium salt was purchased from Shanghai Sangon Biological Engineering Co., Ltd. (Shanghai, China). All cell culture material and chemicals were purchased from Invitrogen (Caelsbad, CA, USA). Acetonitrile and methanol were HPLC-grade purchased from Merck (Kga, Darmstadt, Germany). Deionized water was used throughout the experiments. All other reagents were commercially available and analytical grade.

### Experimental animals

Male healthy Sprague-Dawley rats (210–250 g) were obtained from the experimental animal center of Luye Pharma (Yantai, China) and were housed in rat cages with room temperature (21±1°C) and 50±5% relative humidity. The animals were given a standard pellet diet and water. Rats were fasted 12 h before each experiment with free access to water. Animal experiments were carried out in strict accordance with the Guidelines for Animal Experimentation of Yantai University (Yantai, China) and the protocol was approved by the Animal Ethics Committee of this institution. All surgery was performed under urethane anesthesia, and all efforts were made to minimize suffering.

### Pharmacokinetic studies

To further investigate the pharmacokinetic characteristics of 24R-epimer and 24S-epimer, rats were randomly divided into eight groups with five each. Six groups of rats were intragastrically administered 24R-epimer or 24S-epimer at doses of 5 mg/kg, 10 mg/kg and 20 mg/kg, respectively. Another two groups were intravenously administered 24R-epimer and 24S-epimer at a dose of 1 mg/kg, respectively. Blood samples were collected from the suborbital veniplex at predose and at 0.083, 0.25, 0.5, 1, 2, 3, 4, 6, 8, 12 and 24 h after dosing. Plasma was separated by centrifugation at 5000 g for 10 min and kept at −20°C until analysis. Plasma concentrations of the epimers were determined as described previously [25].

### Cell culture

Caco-2 cells were obtained from the American Type Culture Collection (Manassas, VA), routinely cultured in DMEM supplemented with 10% fetal bovine serum, 1% nonessential amino acids, and 100 U/ml penicillin and streptomycin. The cells were grown in an atmosphere of 5% CO_2_ at 37°C and cell medium was changed every other day. They were passaged upon reaching ∼80% confluence. The cells in this study were between 39∼48 passages. The concentrations of both epimers in cell experiments were measured by LC-MS/MS.

### Cell viability assay

Caco-2 cells were seeded into 96-well plates at a density of 25,000 cells per well. 24R-epimer and 24S-epimer were prepared by dissolving in DMSO and diluting with culture medium at the concentrations of 1–100 µM, respectively. The final concentration of DMSO was 1% (v/v), this volume limitation was abided by strictly in all subsequent experiments. Six parallel wells were prepared for each concentration of each test compound. And 1% DMSO in the culture medium was added as the control. After incubated for 48 h, an MTT solution was added, and the cells were incubated for another 4 h at 37°C. Then removed the supernatant, and 150 µl of DMSO were added to the cells to dissolve the formation. The absorbance at 570 nm was measured with enzyme-labeled instrument (SpectraMax M5, Molecular Device, USA).

### Cellular uptake in Caco-2 cells

Caco-2 cells were seeded in 12-well plates at a density of 100,000 cells per cm^2^ and were incubated to reach confluence. The culture medium was removed and cells were washed with Hank's balanced salt solution (HBSS). Then HBSS containing 24R-epimer (1, 5, 20 µM) or 24S-epimer (1, 5, 20 µM) were added and the cells were incubated for 5, 15, 30, 60, 120, 180 min at 37°C, respectively. Followed incubation, the solution was removed and cells were washed three times with ice-cold HBSS. Then cells were lysed in ice-cold cell lysis buffer for 30 min. After centrifugation, the supernatant was stored at −20°C until analysis. Protein concentrations were measured by BCA protein assay kit (Beyotime Institute of biotechnology, Jiang Su, China). All experiments were conducted in triplicate.

### Transport studies across Caco-2 cell monolayers

Caco-2 cells were seeded on a permeable polycarbonate insert (Millicell cell culture insert, 12 mm diameter, 0.4 µm pore size, Millipore Corporation, German) in 12-well tissue culture plates with a density of 1.0×10^5^ cells per insert. When cultured 17∼21 days after seeding, fluorescein permeability and transepithelial electrical resistance (TEER) measurement (Millicell-ERS epithelial volt-ohm meter, Millipore Corporation, German) were used to evaluate the integrity of the Caco-2 cell monolayers. The apparent permeability coefficient (P_app_) of fluorescein was less than 5×10^−7^ cm/s and the TEER value of the monolayers used in the transport study was more than 600 Ω·cm^2^. Before initiation of transport studies, the cell monolayers were washed with warm HBSS. Then, HBSS containing 24R-epimer (1, 5 and 20 µM) or 24S-epimer (1, 5 and 20 µM) was loaded into either apical or basolateral chambers, in the absence or presence of 10 µM verapamil (P-gp inhibitor) pre-incubated for 1 h. Aliquots from the receiver chamber were collected after incubation for 30, 60, 120 and 180 min at 37°C and added an equal volume of HBSS. The collected solutions were stored at −20°C until analysis. All experiments were conducted in triplicate.

### Effects on cellular retention of rhodamine 123 in Caco-2 cells

Caco-2 cells were seeded in 12-well plates and used for experiments when reached 80% confluences. In brief, cultured cells were washed with warm HBSS, then, preincubated in HBSS containing 24R-epimer (1, 5, 10 µM), 24S-epimer (1, 5, 10 µM) or 1% DMSO (as control) for 1 h. After that, 5 µM rhodamine 123 was added. Verapamil (10 µM) was used as a positive control. After incubation for another 2 h, the solution was transferred and the cells were ringed three times with ice-cold HBSS. Then cells were lysed in ice-cold cell lysis buffer for 30 min. Protein concentrations were measured in the same manner as described earlier. Rhodamine 123 was determined by spectroscopy with enzyme-labeled instrument. The cell lysates were stored at −20°C until analysis. All experiments were conducted in triplicate.

### Effects on P-gp substrate transport of digoxin across Caco-2 cell monolayers

The Caco-2 cell transport model was established in the same manner as described earlier. Then, HBSS containing 24R-epimer (1 and 10 µM), 24S-epimer (1 and 10 µM) or 1% DMSO (as control) was firstly added into both apical and basolateral champers and incubated 1 h at 37°C. Then, 5 µM digoxin was added to either apical or basolateral champer to incubate another 2 h. At the designated time, aliquots from the receiver chamber were taken for analysis. Verapamil (10 µM) was used as a positive control. All experiments were conducted in triplicate. The concentration of digoxin was determined by LC-MS/MS.

### In situ single-pass intestinal perfusion in rats

Rats were anesthetized with urethane by intraperitoneal injection (1.5 g/kg) and placed on a heating surface so as to maintain normal body temperature. The abdomen was opened with a midline incision of 2 to 3 cm and an intestinal segment of approximately 20 cm was cannulated on two ends with plastic tubing. Care was taken to avoid disturbance of the circulatory system and the exposed segment was kept moist with warm normal saline solution. The perfusion intestinal segment was precleaned by passing 10 ml of blank perfusion buffer (Krebs-Ringer buffer, pH 6.8) using an infusion pump at a flow rate of 1.0 ml/min. A solution containing 1 µM rhodamine 123, 2 µg/ml phenol red (negative control) in the absence or presence of 24R-epimer (1, 5, 20 µM), 24S-epimer (1, 5, 20 µM) or verapamil (20 µM, positive control) was perfused at a flow rate of 1.0 ml/min through the intestinal segment for 2 min, then changed to 0.1 ml/min for 120 min. Outlet perfusion samples are collected every 10 min, blood samples were collected at 120 min after perfusion and the plasma were separated. At the end of the experiment, the radius and the length of the intestinal segment were measured. Rhodamine 123 and phenol red were determined using spectroscopy and plasma concentrations of both epimers were analyzed by LC-MS/MS. All experiments were conducted in three rats each group.

### Effects on basal intestinal P-gp expression

After reaching 80% confluences, Caco-2 cells cultured in 6-cm dishes were co-incubated with 24R-epimer (1 and 10 µM), 24S-epimer (1 and 10 µM) or DMSO (1%, v/v). After incubation for 48 and 72 h, the cells were washed with PBS and then lysed in ice-cold cell lysis buffer with 100 µg/ml phenylmethanesulfonyl fluoride (PMSF) for 30 min. The samples collected were centrifuged at 10,000 g for 30 min at 4°C. And the supernatants were stored at −80°C until use. Protein concentrations were measured by a BCA protein assay kit.

Samples were reconstituted in sample loading buffer of SDS-polyacrylamide gel electrophoresis and boiled for 5 min for protein denaturation. Protein samples were separated on a 7.5% SDS-polyacrylamide gel and transferred onto a polyvinylidene difluoride (PVDF) membrane (Millipore Corporation, German). The membrane was blocked after blotting with 5% skim milk in Tris-buffered saline-Tween 20 (TBS-T) for 2 h. Immunoblots were washed (4×5 min) by TBS-T, then, incubated with the anti-P-gp antibody C219 (1∶500; Millipore Corporation, German) or anti-β-actin antibody (1∶1000; Bioword Technology, St. Louis Park, MN) for 12 h at 4°C. After that, the membrane was washed (4×5 min) again, incubated with the secondary antibody horseradish peroxidase-conjugated goat anti-mouse lgG (1∶1000; Beyotime Institute of Biotechnology, Jiangsu, China) for 2 h at room temperature, and then washed four times with TBS-T. The membrane was processed with enhanced chemiluminescence (ECL) detection reagents, and then exposed to photographic films for visualization of the signal. The β-actin levels were determined by Western blotting to correct the P-gp protein band intensity.

### Analytical methodology

24R-epimer, 24S-epimer and digoxin were quantified by LC-MS/MS. The system consisted of an 1100 series HPLC system (Agilent Technologies, Waldbronn, USA) and a TSQ Quantum Access tandem mass spectrometer (Thermo Electron Corporation, San Josem, CA, USA) with an electrospray ionization source. Selective reaction monitoring (SRM) in the positive ionization mode was applied to quantity. The analytical columns were a Shim-pack XR-ODS C18 column (50 mm×2.1 mm i.d., 2.2 µm, Shimadzu, Japan). The rat plasma samples, cell lysates and buffer solutions were all prepared by liquid–liquid extraction. Detailed extraction solvents, LC conditions and mass parameters were shown in Table 1.

**Table 1:** Analytical conditions in LC-MS/MS analysis of 24R-epimer, 24S-epimer and digoxin.

Compound	SRM (m/z)	CID (eV)	Extraction solvent	Mobile phrase
24R-epimer	477.2→441.2,	20	n-hexane-dichloromethane-	Acetonitrile-water (containing 0.2% formic
24S-epimer	477.2→441.2,	20	isopropanol (2∶1∶0.1, v/v/v)	acid) (80∶20, v/v)
Tanshinone IIA (IS)	295.0→249.0,	28		
Digoxin	781.3→355.1,	26	ethyl acetate	Methanol-water (containing 0.4% acetic acid
Phenacetin (IS)	180.3→110.3,	26		and 0.4 mM ammonium acetate) (75∶25, v/v)

CID, collision induced decomposition voltage; IS, internal standard.

Rhodamine 123 and phenol red in cell lysates or intestinal perfusion buffers were quantified by spectroscopy using enzyme-labeled instrument (SpectraMax M5,Molecular Device, USA). An aliquot of 200 µl of sample was added in 96-well plates, the excitation and emission maxima for rhodamine 123 were 485 and 535 nm, respectively. The detection wavelength of phenol red was 562 nm.

### Data analysis

The pharmacokinetic parameters of 24R-epimer and 24S-epimer in rats were obtained by noncompartmental analysis using WinNonlin Version 6.3 (Pharsight Corporation, USA). For the transport assay, the apparent permeability coefficient (P_app_) was calculated as in eq. 1: 
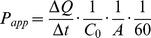
(1)


ΔQ is the transport quantity (nanomoles), Δt is the time of transport (second), C_0_ is the initial concentration in the donor chamber, and A is the surface area of the membrane (centimeters squared). The efflux ratio (ER) was calculated as in eq. 2: 
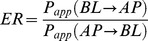
(2)


In the single-pass perfusion experiment, the absorption rate constant k_a_ and drug permeability across rat ileum (P_eff_) were calculated as in eq. 3 and 4. 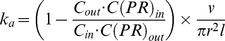
(3)

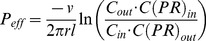
(4)


C_out_ is the outlet concentration of rhodamine 123 at specific time interval, C_in_ is the inlet concentration of rhodamine 123, C(PR)_in_ and C(PR)_out_ are the inlet and outlet concentration of phenol red, respectively, *v* is the flow rate through the ileum segment, *r* is the radius of the ileum, and *l* is the length of perfused segment.

The data were expressed as mean ± S.D. Pearson correlation analyses and Student's *t* test were used to analyze data. The difference was considered to be statistically significant if the probability value was less than 0.05 (*p*<0.05).

## Results

### Pharmacokinetic characteristics

**Figure 2:**
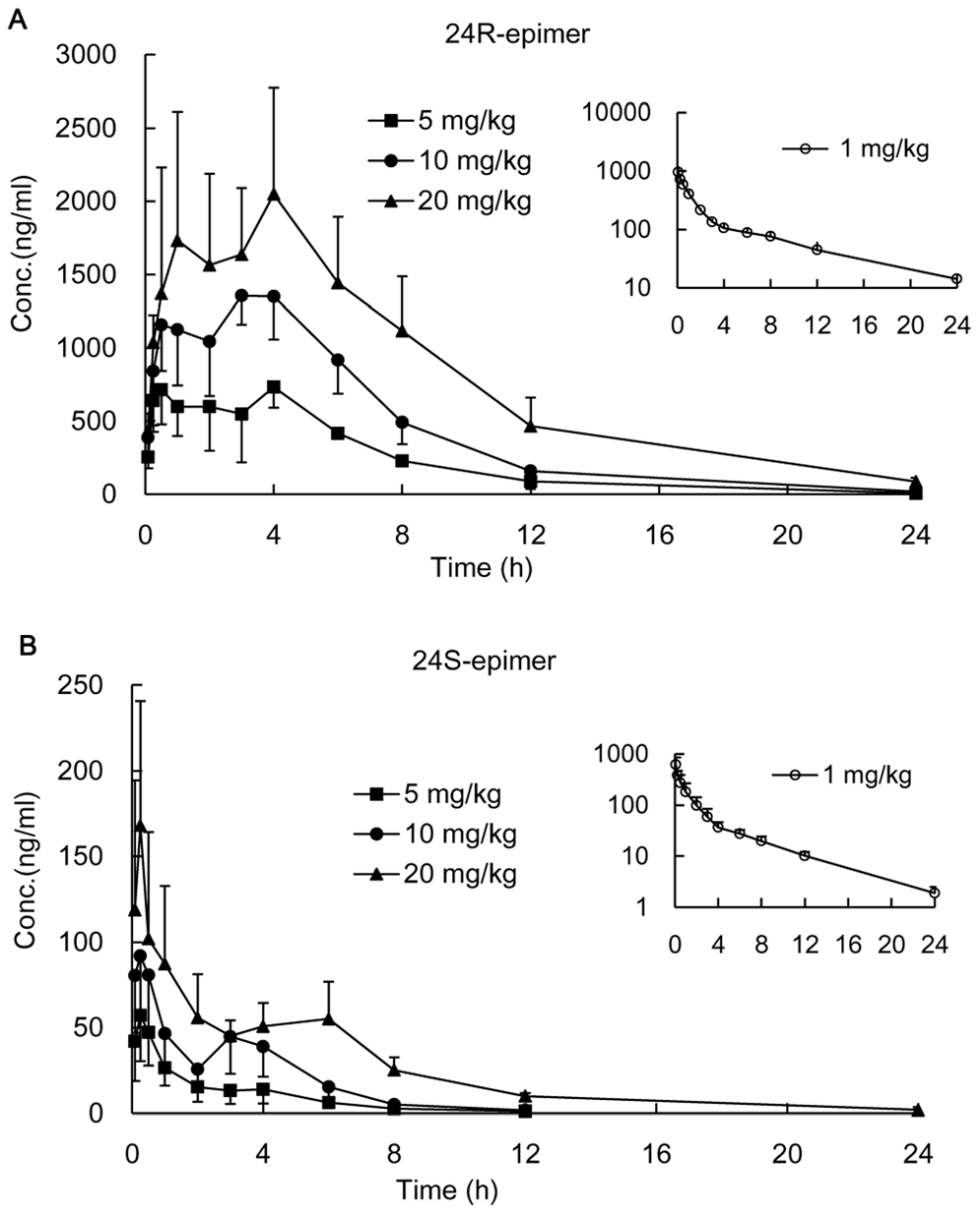
Plasma concentration-time profiles of 24R-epimer and 24S-epimer in rats. The intragastric administration groups were administered 24R-epimer (A) or 24S-epimer (B) at doses of 5, 10 and 20 mg/kg, respectively. The intravenous groups were presented in smaller figure administered 1 mg/kg of 24R-epimer or 24S-epimer, respectively. n = 5 per group.

The mean plasma concentration-time profiles of 24R-epimer or 24S-epimer in rats after oral administration or intravenous injection were shown in Fig. 2. The pharmacokinetic parameters were listed in Table 2. The dose ratio of oral administration was 1∶2∶4, and the ratio of the mean areas under curve (AUC_(0-t)_) was 1∶2.1∶3.7 for 24R-epimer and 1∶2.1∶4.8 for 24S-epimer, respectively. This showed a linear kinetic characteristic within the dosing range of 5–20 mg/kg for both epimers, and correlations were significant (*p*<0.05). The AUC_(0-t)_ of 24R-epimer were 30.7–39.6 folds higher than those of 24S-epimer. Although the results displayed the greater difference on the AUC value compared with our previous study results [25], the difference for each epimer between two experiments were acceptable (the AUC ratio was 80.8%∼118.7%, the C_max_ ratio was 82.3%∼128.6%). The mean retention time (MRT) of 24R-epimer was significantly longer than that of 24S-epimer (*p*<0.05). Absolute bioavailability was determined from the ratio of dose normalized AUC values obtained from oral versus intravenous administration. The absolute bioavailability was 43.01–46.50% for 24R-epimer and 3.06–3.61% for 24S-epimer in rats, respectively.

**Table 2:** Pharmacokinetic parameters of 24R-epimer and 24S-epimer in rat plasma after oral administration and intravenous administration of 24R-epimer or 24S-epimer, respectively.

	Dose mg/kg	T_max_ h	C_max_ ng/ml	AUC_(0-t)_ ng/ml•h	AUC_(0-∞)_ ng/ml•h	MRT_(0-t)_ h	t_1/2_ h	BA (%)
24R-epimer	5	2.7±1.5	911.8±119.0	5094.1±240.6	5407.3±230.5	5.2±1.2	2.3±0.6	46.50
	10	3.0±1.2	1498.1±147.9	10627.0±369.5	10699.2±377.3	5.3±0.8	2.9±0.8	48.50
	20	3.6±1.8	2339.9±647.8	18849.0±2630.2	19184.2±2456.3	6.6±1.0	3.7±1.3	43.01
	1 (iv)	0.083±0	953.4±249.3	2191.1±153.4	2325.0±161.6	5.1±0.8	6.5±1.6	
24S-epimer	5	0.65±0.76	63.3±21.2	128.8±29.6	130.0±31.0	2.7±0.6	2.4±1.1	3.06
	10	0.90±1.18	106.0±21.7	271.9±54.0	275.5±52.8	2.9±0.7	1.9±0.7	3.23
	20	0.60±0.78	198.9±92.1	607.6±57.4	614.7±56.4	5.2±0.6	3.2±0.4	3.61
	1 (iv)	0.083±0	618.1±241.1	841.9±238.4	855.3±232.4	3.5±0.6	4.7±0.8	

(n = 5 per group).

iv, intravenous group; BA, bioavailability.

### Cell viability assay

**Figure 3:**
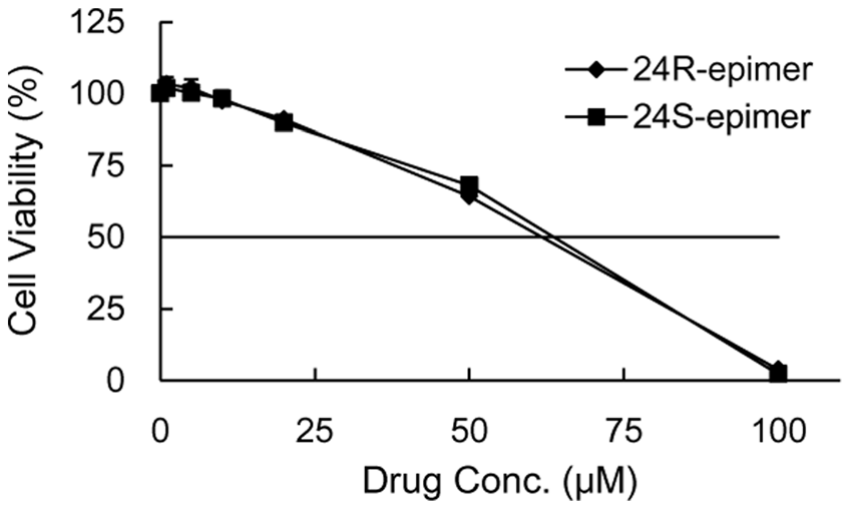
The effect of 24R-epimer and 24S-epimer on Caco-2 cell viability. Cells were incubated with 24R-epimer or 24S-epimer for 48 h at the concentration range of 1–100 µM, respectively; and the cell viability was measured by MTT assay. Data are the mean ± S.E. of three independent experiments.

24R-epimer or 24S-epimer incubated with Caco-2 cells for 48 h didn't cause obvious cell toxicity at concentrations of 1–20 µM, and cell survival rate was above 90% compared with the control when drug concentrations were not more than 20 µM. As shown in Fig. 3, the cytotoxic effects of the two epimers on the Caco-2 cells had not significant differences. This indicated it was suitable to carry out subsequent cell experiments at the concentration range of 1–20 µM for both epimers.

### Cellular uptake in Caco-2 cells

**Figure 4:**
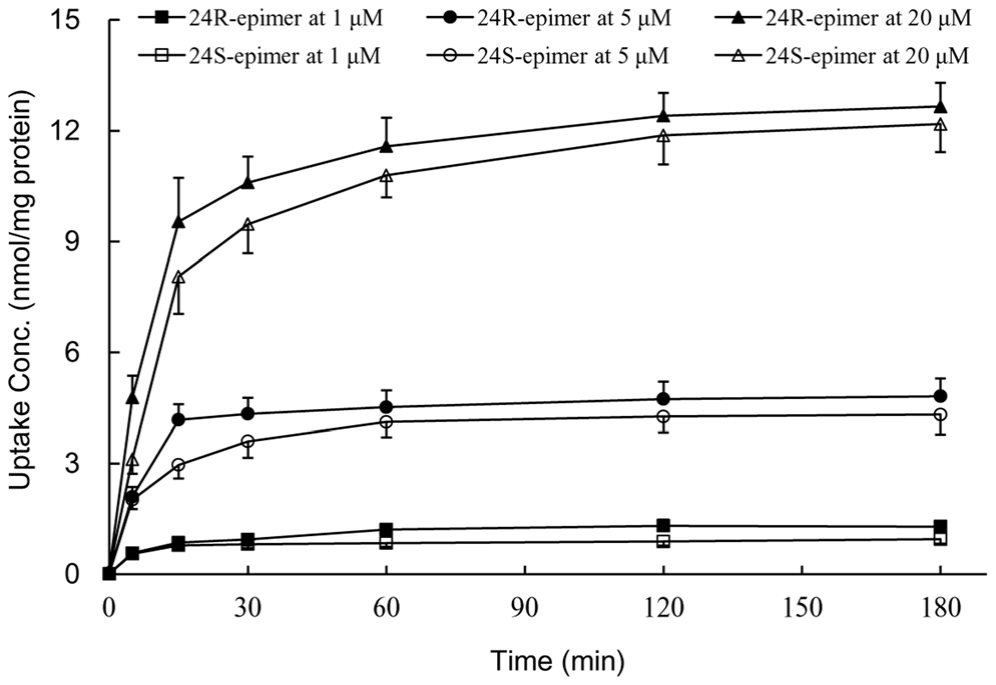
The uptake concentration-time profiles of 24R-epimer and 24S-epimer in Caco-2 cells. 24R-epimer and 24S-epimer concentrations: 1, 5 and 20 µM, respectively. Data are the mean ± S.E. of three independent experiments.

Cellular uptake concentration-time curves of both epimers were shown in Fig. 4. The intracellular concentrations increased sharply in the first 15 min at low, middle and high concentrations (1, 5, 20 µM), and reached platform at 2 h. Moreover, the uptake of 24R-epimer was slight faster and higher than 24S-epimer, the intracellular exposure amount of 24R-epimer was about 1.2 folds greater than that of 24S-epimer. Furthermore, the intake of 24R-epimer and 24S-epimer was linearly increased within the concentration range of 1–20 µM (*p*<0.05), indicating passive diffusion could be one of transmembrane pathways for both epimers. Therefore, the incubation time and concentrations were set for 2–3 h and 1–20 µM in subsequent cell experiments, respectively.

### Transport studies across Caco-2 cell monolayers

The P_app_ values of 24R-epimer and 24S-epimer in both AP→BL and BL→AP directions were shown in Table 3, which demonstrated significant difference between 24R-epimer and 24S-epimer in the ability of transportation across Caco-2 cell monolayers. 24R-epimer has better transmembrane permeability with the P_app_ values 5–7 folds higher than that of 24S-epimer at concentrations of 1, 5 and 20 µM. Furthermore, the efflux ratio of 24R-epimer was approximately 1 in the absence or presence of verapamil. While that of 24S-epimer decreased by 1 fold after preincubated with verapamil. The above results demonstrated 24R-epimer was not a substrate of P-gp, but 24S-epimer was.

**Table 3:** The apparent permeability (P_app_) and efflux ratio (ER) of 24R-epimer and 24S-epimer across Caco-2 monolayers.

	Conc. (µM)	P_app_ (×10^−7^ cm/s)		ER
		AP→BL	BL→AP	
24R-epimer	1	74.3±9.3	81.2±6.0	1.1
	5	71.7±4.7	72.5±5.1	1.0
	20	81.0±13.2	84.6±7.5	1.0
	20+ verapamil	78.5±9.2	89.2±9.0	1.1
24S-epimer	1	10.5±1.5	15.7±1.2	1.5
	5	13.6±2.1	21.1±1.6	1.6
	20	13.9±0.4	25.4±1.5	1.8
	20+ verapamil	14.1±1.1	13.0±0.8	0.9

Data are the mean ± S.E. of three independent experiments.

### Effects on the cellular retention of rhodamine 123 in Caco-2 cells

**Figure 5:**
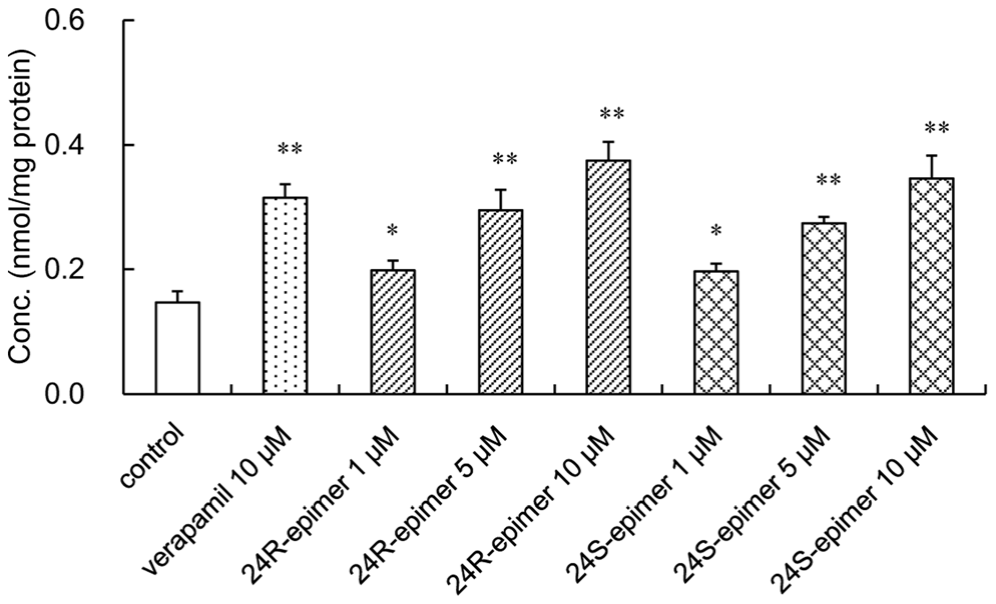
Effects of 24R-epimer and 24S-epimer on the accumulation of rhodamine 123 in Caco-2 cells. Cells were preincubated for 1-epimer or 24S-epimer at concentrations of 1, 5 and 10 µM, respectively; and followed by co-incubation for 2 h in the presence of 5 µM rhodamine 123. Data are the mean ± S.E. of three independent experiments. ^*^ *p*<0.05 versus control; ^**^ *p*<0.01 versus control.

As shown in Fig. 5, 24R-epimer and 24S-epimer could increase cellular accumulation of rhodamine 123, a classic substrate of P-gp, in Caco-2 cells. Verapamil, as a positive inhibitor of P-gp, resulted in a significant increase of intracellular accumulation of rhodamine 123 by 2.1-fold. And the accumulation of rhodamine 123 was significantly increased by 1.4–2.6 folds in the presence of 24R-epimer and 1.3 to 2.4 folds in the presence of 24S-epimer at the concentration range of 1–10 µM. This indicated that both 24R-epimer and 24S-epimer might be an inhibitor of P-gp, and their inhibitory abilities were comparative at the same concentration level.

### Effects on P-gp functions in Caco-2 cells

**Figure 6:**
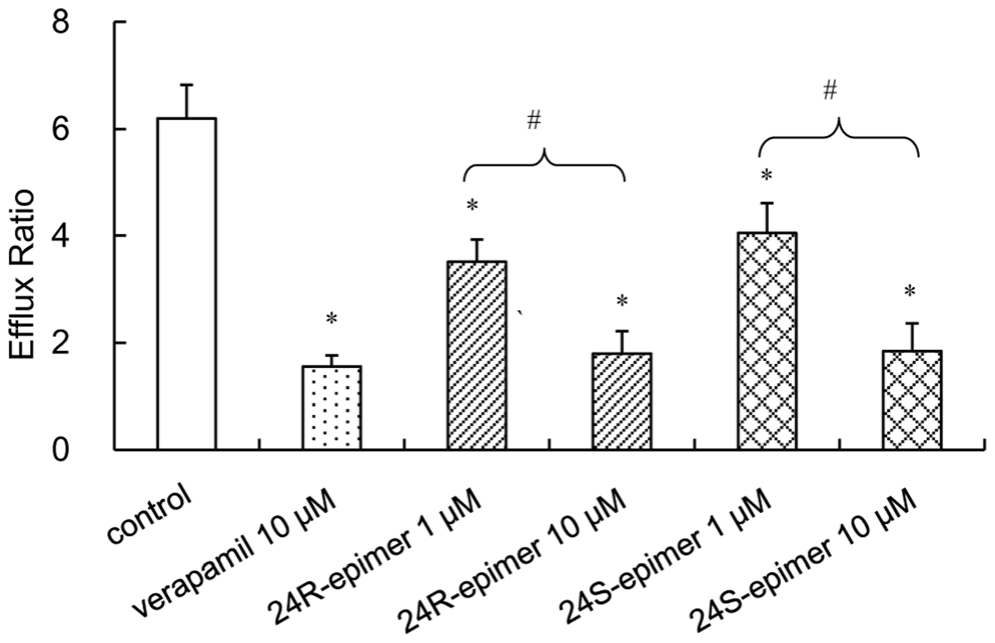
Effects of 24R-epimer and 24S-epimer on the transport of P-gp substrate digoxin across Caco-2 cell monolayers. Cells were preincubated for 1-epimer (1 and 10 µM) or 24S-epimer (1 and 10 µM), and followed by co-incubation for 2 h in the presence of 5 µM digoxin. Data are the mean ± S.E. of three independent experiments. ^*^ *p*<0.01 versus control, ^#^ *p*<0.01 between groups.

Digoxin, as a substrate of P-gp, exhibited highly polarized transport across Caco-2 cell monolayers with high efflux ratios. As shown in Fig. 6, 24R-epimer or 24S-epimer significantly decreased the efflux ratio of digoxin at 1 µM and 10 µM (*p*<0.01). And the inhibition at 10 µM was obviously stronger than that of at 1 µM (*p*<0.01). Furthermore, the inhibitory effects were no significant difference between verapamil, 24R-epimer and 24S-epimer at a 10 µM concentration.

### Effects on rhodamine 123 in situ single-pass intestinal perfusion model in rats

In situ single-pass intestinal perfusion of rhodamine 123 in the presence or absence of 24R-epimer, 24S-epimer or verapamil were performed. The intestinal permeability and absorption rate constant of rhodamine 123 were calculated and shown in Table 4. The intestinal permeability and absorption rate constants of rhodamine 123 were significantly increased by 24R-epimer and 24S-epimer at concentrations of 5 and 20 µM (*p*<0.01), and the effect of 24R-epimer was significant stronger than that of 24S-epimer (*p*<0.05 at 5 µM, *p*<0.01 at 20 µM). After perfusion for 2 h, the plasma concentrations of 24R-epimer could be detected at low, middle and high concentration groups and increased in a concentration-dependent manner; but that of 24S-epimer was not available. These differences might result from the poor membrane permeability of 24S-epimer and strong first pass effects on 24S-epimer. It needs to further investigate the metabolic differences between 24-epimer and 24S-epimer.

**Table 4:** Effects of 24R-epimer and 24S-epimer on absorption rate constant (*k*_a_), permeability (P_eff_) of rhodamine 123 in the single-pass intestinal perfusion model in rats, and the plasma concentration of both epimers after perfusion for 2 h.

	Conc.	Rhodamine 123		Plasma Conc.
	(µM)	*k*_a_ (×10^−2^/min)	P_eff_ (×10^−5^ cm/s)	(ng/ml)
Control (1% DMSO)		1.19±0.08	1.64±0.12	
24R-epimer	1	1.44±0.15	2.02±0.24	5.92±1.58
	5	2.01±0.12* #	2.93±0.19* #	26.66±5.29
	20	2.96±0.13* ##	4.92±0.21* ##	95.25±23.56
24S-epimer	1	1.17±0.13	1.62±0.22	ND
	5	1.65±0.12*	2.33±0.14*	ND
	20	1.93±0.09*	2.87±0.24*	ND
Verapamil	20	3.35±0.15*	5.72±0.29*	

(n = 3 per group).

ND, not detected;

^*^ *p*<0.01 versus control;

# *p*<0.05 versus 24S-epimer at 5 µM concentration;

## *p*<0.01 versus 24S-epimer at 20 µM concentration.

### Effects on basal intestinal P-gp expression

**Figure 7:**
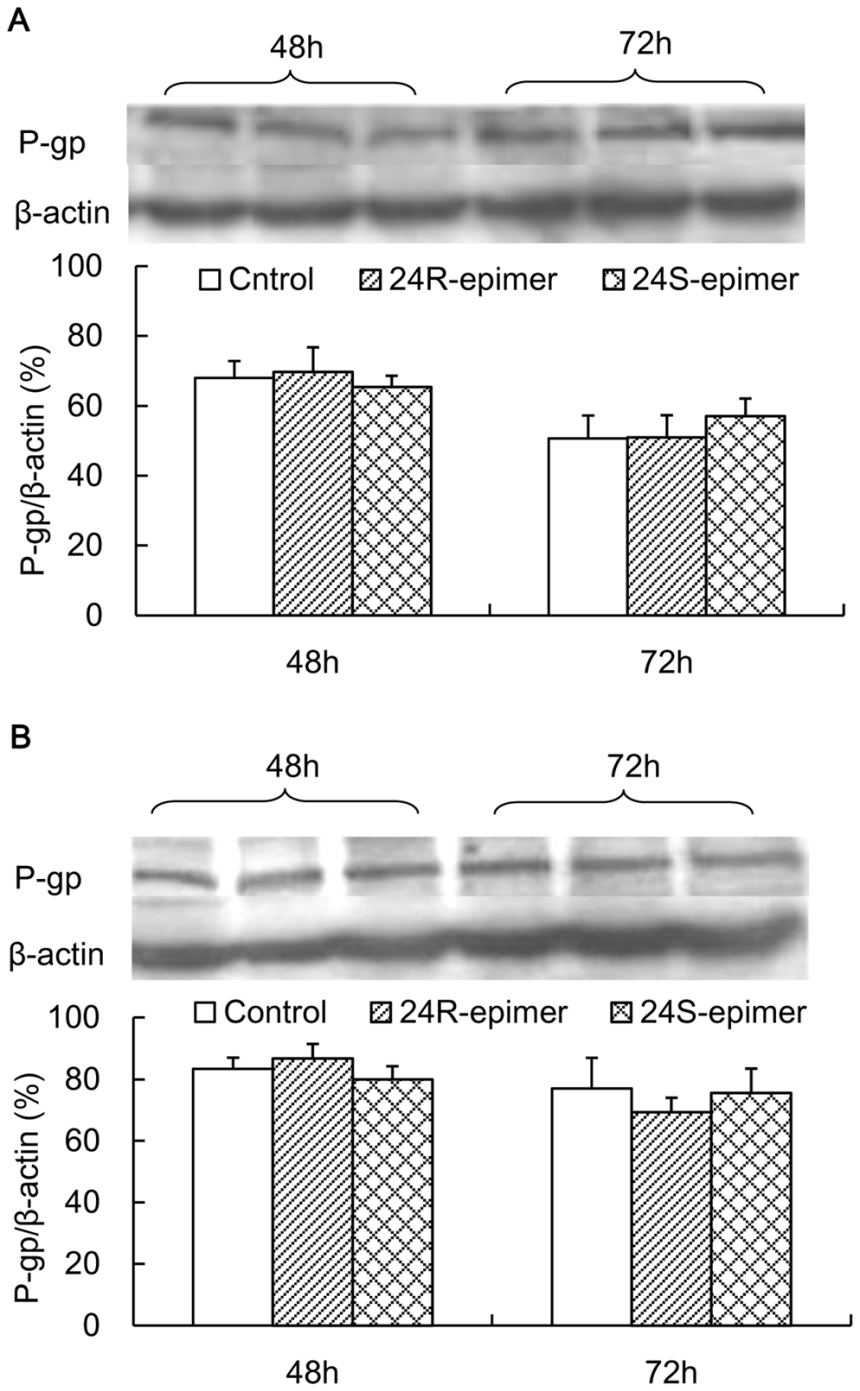
Effects of 24R-epimer and 24S-epimer on P-gp expression levels in Caco-2 cells. Cells were exposed to 24R-epimer or 24S-epimer at concentrations of 1 µM (A) and 10 µM (B), and 1% of DMSO as a solvent control for 48 and 72 h. The bar graphs show the P-gp protein band intensity corrected by that of β-actin.

After Caco-2 cells were treated with 24R-epimer or 24S-epimer for 48 h or 72 h, the lysates of cells were recovered and subjected to Western blot analysis. As shown in Fig. 7, both 24R-epimer and 24-epimer had no significant effect on P-gp expression at 1–10 µM concentration.

## Discussion

Stereochemistry is an important dimension in pharmacology and it often results in different pharmacological activities and stereoselective pharmacokinetics of chiral xenobiotics. It has been an area of neglected dimensionality until relatively recently [30]. Many chiral drugs, including enantiomers and epimers, have little information on stereoselective behavior *in vivo*, especially medicine herbs. 20(S)-protopanaxadiol ocotillol type epimers, 24R-epimer and 24S-epimer, were the main metabolites of PPD. In the present study, we studied the pharmacokinetics and absolute bioavailability of both epimers after intragastrically administration at three different dosages, to further explore their pharmacokinetic characteristics. The results obtained should provide valuable information for understanding the stereoselective pharmacokinetics of 24R-epimer and 24S-epimer. After oral administration, it showed a linear kinetic characteristic within the dosing range of 5–20 mg/kg for both epimers according to AUC values. As to C_max_, it could be seen from Fig. 2 that the mean plasma concentrations of 24R-epimer were basically steady at the period of 0.5–4 h after administration at each dosage group, and 24R-epimer showed the more obvious double peaks than 24S-epimer in the concentration-time profiles. In fact, the absorption rate of 24R-epimer was faster than that of 24S-epimer, it basically could reach the maximum absorption concentrations at 0.5 h or 1 h after administration. At the time point, the mean C_max_ was 715.7, 1207.4 and 2111.0 ng/ml at low, middle and high dosages, respectively. This also showed a linear kinetic characteristic between the dosage and C_max_ for 24R-epimer, and correlations were significant (p<0.05). But the appearance of the double peaks at 4 h might be resulted from hepato enteric circulation that made the epimers be reabsorbed into systemic circulation and delay their elimination *in vivo*, which affected the linear relation between the C_max_ and dosages. As to 24S-epimer, the appearance of the double peaks was weaker than that of 24R-epimer, it showed a linear correlation between the C_max_ and dosages (p<0.05). In addition, the absolute bioavailability of 24R-epimer was about 14-fold higher than that of 24S-epimer. Zhang *et al* reported that the C_max_ and AUC of 20(S)-Rh2 were 15-fold and 10-fold higher than those of 20(R)-Rh2, respectively, with the same dosage for oral administration [23]. Bae *et al* reported the 20S-configuration for both Rg3 and Rh2 exhibited significantly higher plasma concentrations than the one with 20R-configuration [22]. These pharmacokinetic differences were caused by the C20 stereoisomerism, and showed the ginsenosides with S-configuration had higher absorption than the one with R-configuration. However, interestingly, 20(S)-protopanaxadiol ocotillol type epimers are on basis of C20 S-configuration, C24 stereoisomerism resulted in the significant difference in the pharmacokinetics and C24 R-configuration had higher absorption. It was thus evident that biomacromolecules *in vivo* has susceptible recognition to stereo-configuration of micromolecule xenobiotics.

The cytotoxic effects of the two epimers on the Caco-2 cells had not significant differences. Compared with PPD (IC50: 29.8 µM), both epimers showed low cytotoxic potency to the Caco-2 cell line. The study [31] indicated that 20S-24R-epoxy ring reduced activity on growth inhibition of cancer cells for 20(S)-protopanaxatriol metabolites. Owing to the stereoselectivity, the apparent permeability coefficient (the P_app_ value in the absorptive direction) of 24R-epimer (8×10^−6^ cm/s) was about 6 folds higher than that of 24S-epimer (1.3×10^−6^ cm/s). Gu *et al* reported that the permeability difference between 20(R)-Rh2 (2×10^−8^ cm/s) and 20(S)-Rh2 (30×10^−8^ cm/s) was 15 folds [32]. And unlike C20 S-configuration of Rh2, C24 R-configuration had better membrane permeability than S-configuration. In addition, from Rh2 to 20,24-epoxides of PPD, with the increase of lipophilicify, the membrane permeability coefficient increased. In addition, the experiments *in vitro* and *in vivo* all indicated that both 24R-epimer and 24S-epimer had inhibitory effects on P-gp, and the efflux ratio of 24S-epimer was only 1.5–1.8 at concentrations of 1–20 µM. Therefore, as an inhibitor of P-gp, 24S-epimer might inhibit itself efflux. Based on these experimental results, it could be supposed that the stereoselectivity of the C24 epimers in pharmacokinetics was mainly due to S-configuration processed the poor transmembrane permeability and could be distinguished by the P-gp. Western blot analysis for the P-gp demonstrated that both epimers had no action on basal P-gp expression in Caco-2 cells after co-culture for 48 and 72 h. The study [28] also demonstrated that 20(S)-Rh2 had no effect on basal P-gp expression in Caco-2 cells. Hence, the ocotillol type side chain and C24 stereo-configuration did not change the effect of parent drug on expression of P-gp. Sharing the dammarane skeleton, PPD type and ocotillol type ginsenosides had similar effects on P-gp.

Drug-drug interaction (DDI) is a universal phenomenon. On the one hand, DDI should be avoided because it might cause severe clinical side effects. On the other hand, a DDI might benefit a patient by producing an enhanced effect from increased systemic levels of the target drug [33], [34]. An update on the clinical strategy to overcome multidrug resistance in cancer is to inhibit the P-gp [35]–[37]. Kwak *et al* [38] reported selective inhibition of MDR1 by HM30181 increased oral bioavailability and therapeutic efficacy of paclitaxel. Zhang *et al* reported that both 20R and 20S epimers of Rh2 and PPD were inhibitor of P-gp [23]. The synergistically enhancing effect of Rh2 on anticancer therapies has already been reported [39], [40]. Zhao *et al* [29] reported that PPD may be a potential new P-gp inhibitor for cancer treatment in addition to its pro-apoptotic nature. In the present study, 24R-epimer and 24S-epimer, the main metabolites of PPD, were the inhibitors of P-gp *in vitro* and *in vivo*, so both epimers could be used as an intestinal absorption enhancer for P-gp substrate drugs on the basis of a drug-drug interaction. Especially, 24R-epimer had better oral bioavailability and low metabolism burden on organism compared with parent compounds PPD and Rh2.

Although more attention was paid to the P-gp transporter with regard to the stereoselective interaction with transporters, multidrug resistance-associated protein 2 (MRP2, ABCC2) and breast cancer resistance protein (BCRP, ABCG2) also are main extrusion transporters of the ABC transporter family. Some studies investigated the stereoselective interaction between stereoisomers and MRP or BCRP [41]–[43]. Moreover, Kawase *et al* reported that ginsenoside Rg2, unlike others ginsenosides Rb1, Rc, Rd and Rg3, enhanced MRP2 mRNA levels [44]. Jin *et al* reported that ginsenosides PPD, Rh2 and PPT were the potent inhibitors of BCRP, with a rank order of potency PPD>Rh2>PPT, but ginsenosides Rg3, Rg1 and Rh1 were ineffective [45]. The site of attachment of hydroxyl groups and sugar moiety at the steroid skeleton has been shown to influence their biological activities. These all hint us to explore the stereoselective interaction and chiral discrimination between the epimers and MRP2 or BCRP in the follow-up studies.

In conclusion, 24R and 24S epimers, the main metabolites of PPD all showed a linear kinetic characteristic within the dosing range of 5–20 mg/kg, and the absolute bioavailability of 24R epimer was 14-fold higher than that of 24S-epimer. The C24 stereo-configuration resulted in stereoselective absorption differences between 24R-epimer and 24S-epimer; C24 S-configuration processed the poor transmembrane permeability and could be distinguished by the P-gp. Similar to the parent drugs of PPD and Rh2, both 24R-epimer and 24S-epimer were the potent inhibitors of P-gp, and had no effect on P-gp expression in Caco-2 cells. This indicated the inhibitory ability to P-gp was mainly attributed to the dammarane skeleton structure that was the recognize site of P-gp, while the ocotillol type side chain and C24 stereo-configuration didn't affect the action to P-gp.
